# Preliminary Investigation into *Plasmodium*-like Piroplasms (*Babesia*/*Theileria*) among Cattle, Dogs and Humans in A Malaria-Endemic, Resource-Limited Sub-Saharan African City

**DOI:** 10.3390/medsci10010010

**Published:** 2022-02-03

**Authors:** Patrick F. Ayeh-Kumi, Irene A. Owusu, Patience B. Tetteh-Quarcoo, Nicholas T. K. D. Dayie, Kevin Kofi Adutwum-Ofosu, Seth K. Amponsah, Emilia A. Udofia, Emmanuel Afutu, Simon K. Attah, Robert Armah, Robert Aryee, Fleischer C. N. Kotey, Benjamin P. Niriwa, Japheth A. Opintan, Eric S. Donkor, John Ahenkorah

**Affiliations:** 1 Department of Medical Microbiology, University of Ghana Medical School, Accra P.O. Box KB 4236, Ghana; pfayeh-kumi@ug.edu.gh (P.F.A.-K.); ireneamoakohowusu@gmail.com (I.A.O.); ntkddayie@ug.edu.gh (N.T.K.D.D.); skwakuattah@yahoo.com (S.K.A.); slarity@yahoo.com (R.A.); bobby200055@gmail.com (R.A.); fcnkotey@flerholiferesearch.com (F.C.N.K.); pullebenjamin@gmail.com (B.P.N.); jaopintan@ug.edu.gh (J.A.O.); ericsdon@hotmail.com (E.S.D.); 2 Department of Anatomy, University of Ghana Medical School, Accra P.O. Box KB 4236, Ghana; kadutwum-ofosu@ug.edu.gh; 3 Department of Medical Pharmacology, University of Ghana Medical School, Accra P.O. Box KB 4236, Ghana; sethicom@yahoo.com; 4 Department of Community Health, University of Ghana Medical School, Accra P.O. Box KB 4236, Ghana; eudofia@ug.edu.gh; 5 FleRhoLife Research Consult, P.O. Box TS 853, Teshie, Accra, Ghana; 6 College of Health—Yamfo, P.O. Box 23, Ahafo Region, Ghana

**Keywords:** *Babesia*, babesiosis, cattle, dogs, *P. falciparum*, *Theileria*

## Abstract

*Babesia* and *Theileria* are protozoan parasites belonging to the order piroplasmida, transmitted by hard ticks, and can cause diseases known as piroplasmosis. Human infections are usually asymptomatic, except in immuno-compromised persons who present malaria-like symptoms. Moreover, microscopically, the morphologies of *Babesia* and *Theileria* can resemble that of the malaria parasite, *Plasmodium*. In malaria-endemic areas with limited resources, these similarities can increase the possibility of misdiagnosing a patient as having malaria instead of piroplasmosis, which may further lead to inappropriate choice of disease management. This preliminary investigation aimed at detecting *Babesia*/*Theileria* in cattle, dogs and humans in some parts of Accra. Whole blood samples were taken from febrile cattle (*n* = 30) and dogs (*n* = 33), as well as humans diagnosed with malaria (*n* = 150). Blood samples of all study subjects were microscopically screened for possible presence of *haemoparasites*. Samples whose smears had features suggestive of possible piroplasmic infection were all given the label “suspected *Babesia/Theileria*-infected” samples. Nested polymerase chain reaction (PCR) was performed on extracted deoxyribonucelic acid (DNA) from all the “suspected” samples of cattle, dogs and humans, with primer sets that can detect 18S rRNA genes of *Babesia*/*Theileria* spp. In addition to this, amplification was performed on the “suspected” dog samples using the BcW-A/BcW-B primer set which detects the 18S rRNA genes of *B. canis*, while the BoF/BoR primer set which targets the rap-1 region of *B. bovis* and another primer set which detects the 18S rRNA genes of most bovine *Babesia* spp. (including *B. divergens*) were used on the suspected cattle samples. For the human samples, however, additional amplification was done on the extracted DNA using primers for the three other *Babesia* targeted (*B. divergens*, *B. bovis* and *B. canis*). Microscopy showed possible *Babesia*/*Theileria* infection suspected in all three groups of subjects in the following proportions: cattle (10/30; 33%), dogs (3/33; 9%) and humans (6/150; 4%). DNA from one-third of the “suspected” dog samples yielded amplification with *Babesia canis* primers. Moreover, a broad-detecting set of primers (that can amplify some *Babesia* and *Theileria* species) amplified DNA from nine (9/30; 30%) of the “suspected” cattle samples, but none from those of the humans. Although for this study conducted in the city, the *Babesia*/*Theileria* primers used did not amplify DNA from the six “suspected” human samples; the possibility of *Babesia*/*Theileria* infection in humans in other parts of the country cannot be overruled. There is therefore a need for further studies on possible emergence of human babesiosis/theileriosis in other parts of Ghana and sequencing for specific identification of any circulating strain.

## 1. Introduction

Piroplasmosis is an emerging tick-transmitted disease that occasionally infects humans, and is primarily caused by protozoan parasites of the genera *Babesia* and *Theileria* [1,2]. Symptoms of the infection in humans, if any, usually develop within one to nine weeks after exposure, especially in immuno-compromised people, and could range from subclinical to severe disease [3,4]. Clinical manifestations include haemolytic anaemia and nonspecific flu-like symptoms (fever, chills, body aches, weakness, fatigue, etc.,), but some patients risk having splenomegaly, hepatomegaly, jaundice, blood pressure instability and myocardial infarctions in severe cases [5,6,7]. The trophozoite stages of *Babesia* and *Theileria* appear as ring forms, and measure about 1.0 to 5.0 μm; they are non-motile cytoplasmic rings with chromatin dots as nuclei, and closely resemble *Plasmodium falciparum*. There are over one hundred *Babesia* species known, but about five of these (*Babesia microti*, *Babesia duncani*, *Babesia divergens*, *Babesia bovis* and *Babesia venatorum*) have been implicated in human infections [8]. Furthermore, *Theileria* species, such as *Theileria velifera*, *Theileria mutans*, etc., are known to infect cattle [9]. It is noteworthy that some species of *Babesia* are genetically closely related to some *Theileria*, such that primers designed for some *Babesia* are able to amplify some *Theileria* [10,11]. Hence in this study, the rendering “*Babesia*/*Theileria*” has been adapted to represent species of these two genera that share this close genetic relationship.

The symptoms of babesiosis/theileriosis are similar to that of malaria, and the morphology of *P. falciparum* is similar to that of *Babesia* sp. and *Theileria* sp. [7]. Therefore, accurate diagnosis of babesiosis/theileriosis remains a challenge in malaria-endemic regions, such as Ghana. There is no documented information on the presence or absence of *Babesia*/*Theileria* species infecting humans in Ghana, although some reports have been made among animals [9,12]. Meanwhile, in the United States of America, Europe and some other parts of the world, *Babesia* infection in humans has been established and documented [3,13,14,15,16,17]. Immuno-competent, asymptomatic carriers of human *Babesia* sp. and/or *Theileria* sp. are likely to donate blood to possibly already immuno-compromised patients. These individuals who receive such blood and blood products will be at a greater risk of infection with *Babesia* and/or *Theileria* sp., which may lead to severe disease. As Ghana is a malaria-endemic region, there is a possibility that piroplasmosis might be misdiagnosed as malaria, even after laboratory investigations. This is a possibility because routine diagnosis of malaria in the country mainly employs microscopic examination of Giemsa-stained, thick blood smears, or rapid diagnostic test kits. The thick smears may not reveal diagnostic features such as the tetrad and paired configurations that differentiate *Plasmodium* from some *Babesia* (such as *B. microti*). Therefore, preparation and examination of thin smears alongside the thick ones can reveal features (such as classical Maltese cross or paired pear-shaped morphology) suggestive of possible *Babesia* infection. A negative result from the rapid test kit (especially types that are made to detect all four major *Plasmodium* species that infect man) is usually assumed to mean no *Plasmodium* infection, even though there have been cases where smears have been positive [18,19]. This discrepancy has brought suspicion as to what those intracellular parasites may be, if not *Plasmodium*. Further investigations about *Babesia* or *Theileria* in such samples (that are negative for rapid test kit, but show intracellular parasites in smears) will be helpful. Moreover, even though molecular methods could be more sensitive than microscopy, it is important to note that in resource-limited environments, molecular techniques are not that handy for routine clinical diagnosis. Since zoonotic *Babesia*/*Theileria* are transmitted by ticks, screening of cattle and dogs could help provide evidence of the infection among these animals. Knowing this can help in the hypothesis of possible zoonotic infection, since animals could be found living in close proximity with humans in most parts of Ghana.

Additionally, some cases of babesiosis found in other parts of the world were not confirmed until there were reviews of blood smear slides from patients who had initially been misdiagnosed as having *P. falciparum* malaria [15,17,20,21]. Therefore, the current study was conducted to investigate the distribution of piroplasmosis in part of the Greater Accra Region of Ghana, among cattle, dogs and humans.

## 2. Materials and Methods

### 2.1. Study Sites and Blood Samples Collection

The study was a cross-sectional one, conducted in parts of the Greater Accra Region of Ghana from June 2015 to April 2016. This region, even though small in size, is the most densely populated of all the regions of the country. Whole blood samples (*n* = 30) were taken from the jugular veins of febrile cattle at the Accra Cattle Market in “Tulaku” within the Ashaiman Municipality. This market is also the main sales point for most cattle that are brought from places outside Accra, especially northern Ghana, Burkina Faso, Mali and Niger. Cattle that presented with body and joint weakness, dermatitis, weight loss, anorexia, or a combination of any of these were considered sick.

Thirty-three blood samples from the cephalic veins of sick dogs visiting the La Veterinary Hospital were also collected. About 2 mL of venous blood sample was taken from each of the selected subjects for screening. In addition, a total of 150 human blood samples were taken from consenting members of a cattle rearing community in the Ashaiman Municipality who were diagnosed with malaria after laboratory examination of Giemsa-stained, thick blood films at the polyclinic as well as other medical facilities/laboratories in the metropolis. All blood samples were collected into well-labelled EDTA tubes and kept in cold boxes, and thereafter transported to the parasitology laboratory of the Department of Medical Microbiology, University of Ghana Medical School, for examination.

### 2.2. Laboratory Procedures

For the blood samples collected, thin smears, alongside thick smears, were made and stained with Giemsa for microscopic examination. During the examination, samples whose smears had features like classical Maltese cross or paired pear-shaped, ringform morphology suggestive of possible piroplasmic infection (such as some *Babesia*, *Theileria*, non-human plasmodia, chromatin dots, and even haemoplasma/anaplasma) were all given the label “suspected *Babesia/Theileria*-infected” samples [7,13,14,15]. These “suspected” samples were selected for DNA extraction and PCR amplification. In the animals (cattle and dogs), these suspected samples were mainly those whose thin smears showed extra/intra-erythrocytic parasites, while in the human samples, slides that showed erythrocytes with haemo-parasites, but did not react with a rapid diagnostic test kit (Clinigen Diagnostics, Tokyo, Japan) for the four major human *Plasmodium* sp., were selected. The rapid diagnostic kit which was designed to detect antigens in whole blood contained a membrane strip pre-coated with two monoclonal antibodies as two separate lines across the test strip. One line had antibodies specific for *P. falciparum* histidine-rich protein-2 (Pf HRP-2), and another line had antibodies that were *Pan* specific to common *Plasmodium* lactate dehydrogenase (pLDH) of *Plasmodium* species (*P. falciparum, P. vivax, P. ovale* and *P. malariae*). Extraction of total DNA from selected blood samples was performed using the QIAamp DNA mini kit (QIAGEN, Hilden, Germany) according to the manufacturer’s instructions.

Nested polymerase chain reaction (PCR) was performed on the extracted DNA from all the “suspected” samples of cattle, dogs and humans, with primer sets that can detect 18S rRNA genes of *Babesia*/*Theileria* spp., namely, primers Bab5 (5′-AATTACCCAATCCTGACACAGG-3′) and Bab8 (5′-TTTGGCAGTAGT TCGTCTTTAACA-3′) for the first round of amplification and primers Bab6 (5′-GACACAGGG AGGTAGTGACAAGA-3′) and Bab7 (5′-CCCAACTGCTCCTATTAACCATTAC-3′) for the second round of PCR (Table 1) [22].

In addition to this, amplification was performed on the “suspected” dog samples, using BcW-A/BcW-B primer set which detects the 18S rRNA genes of *B. canis*, while BoF/BoR primer set which targets the rap-1 region of *B. bovis* and another primer set which detects the 18S rRNA genes of most *Bovine Babesia* spp. (including *B. divergens*) were used on the suspected cattle samples. For the human samples, however, additional amplification was done on the extracted DNA using primers for the three other *Babesia* targeted (*B. divergens*, *B. bovis* and *B. canis*) (Table 1) [22,23,24,25].

All laboratory safety precautions were followed. The reaction solution for PCR contained 12.5 μL of One Taq 2X master mix with GC buffer (New England Biolabs Inc, Ipswich, MA, USA), 0.5 μL of 10 μM of each primer, 3.5 μL of One Taq high GC enhancer and 8 μL of the DNA in a final volume of 25 μL.

The cycling conditions for the nested PCR amplification using the *Babesia/Theileria* primers involved a total of 30 cycles for “nest one”: denaturing at 94 °C for 30 s, annealing at 55 °C for 30 s, extending at 68 °C for 1 min, with an initial pre-incubation at 94 °C for 30 s and a final extension at 68 °C for 10 min. The “nest two” was carried out for 40 cycles with the same cycling conditions as “nest one” except an annealing temperature of 58 °C. Aliquots (5 μL) of all amplified DNA were subjected to electrophoresis using 2% agarose gel, stained with ethidium bromide and detected under UV trans-illumination. The expected target size for the nested amplified product was approximately 400 bp, and the band size was measured using a 100 bp DNA ladder (New England Biolabs Inc, Ipswich, MA, USA). Conditions for the other species primers are summarised in Table 2. DNA amplification was carried out using the available thermo-cycler, an MJ Research PTC-150 MiniCycler (MJ Research Inc., St. Bruno, QC, Canada).

### 2.3. Statistical Analysis

Data obtained from this study were analysed using the Microsoft Office Excel 2007 software (Microsoft^®^ Office Professional 2007, Microsoft Corporation, St. Redmond, WA, USA). Descriptive statistics, such as frequencies and percentages were used in summarising the data collected from the various hosts (cattle, dogs and humans).

## 3. Results

The proportion of the cattle suspected to be infected, based on the microscopic examination, was 33% (10/30), out of which nine were detected by PCR with the *Babesia*/*Theileria* primers, but none (0/10) with the *B. bovis* and *B. divergens* primers (Table 3). With regard to the dogs (*n* = 33), 33.3% (1/3) of the three “suspected” samples, was confirmed positive by PCR, using the primers for *B. canis*, while none (0/3) was amplified with the *Babesia*/*Theileria* primers (Table 3). Moreover, none of the six “suspected” human samples (*n* = 150) was amplified with either of the *Babesia/Theileria*, *B. bovis*, *B. canis*, or *B. divergens* primers (Table 3).

### 3.1. Microscopic Suspicion of Babesia/Theileria in Cattle, Dogs and Humans

**Figure 1:**
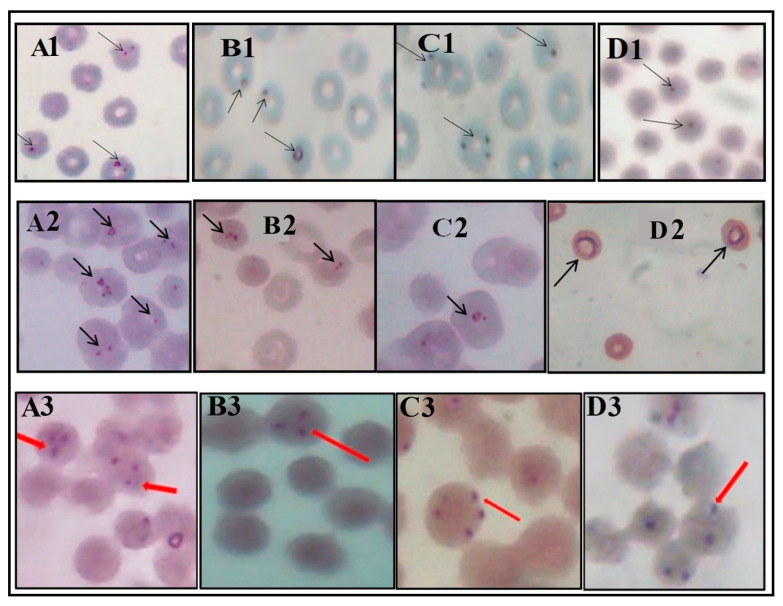
Blood stages of intra-erythrocytic parasites/organisms seen in thin blood films of cattle, dog and human samples. Arrows point to intra-erythrocytic parasites/organisms. **A1**–**D1** shows slides from cattle blood samples with different configurations of parasites/organisms. **A2**–**D2** shows slide from dog blood samples, where **A2**–**C2** are fields from two samples that did not have DNA amplification, using the *B. canis* primers. **A2** and **C2** are different fields of the same slide, and **D2** is a field from the sample that was PCR-amplified with the *B. canis* primers. **A3**–**D3** displays slides from the human blood sample, where Slides **A3** and **B3** show “triad” configuration, **C3** shows “tetrad” configuration, and **D3** shows “paired” configuration.

Intra-erythrocytic parasites were observed in ten of the thirty cattle samples. Most of the infected erythrocytes had one or two parasites infecting them (Figure 1, A1–D1). Slides A1, B1 and C1 are from some of the nine samples that were later confirmed to be infected with *Babesia/Theileria*. Although no tetrad configuration was observed, there were some red blood cells infected with parasites in a triad configuration (Figure 1, C1). Slide D1 showed intra-erythrocytic organisms observed under the microscope, from the single cattle sample that subsequently had no amplification by PCR using *Babesia/Theileria* primers, probably representing *Anaplasma* or something else.

In the dog samples, although most of the infected erythrocytes had one parasite infecting them, some had more than one (Figure 1, A2–D2). Slides A2 to C2 are from the two blood samples that were not PCR-amplified with both *B. canis* and *Babesia/Theileria* primers, although intra-erythrocytic parasites were observed in them. Slide D2 is from the single dog sample that was subsequently PCR-amplified with the *B. canis* primers. A complete intra-erythrocytic ring was observed in that slide.

In the human blood samples, various parasite configurations were observed: the “triad” configuration (Figure 1, Lanes A3 & B3), tetrad configuration (Figure 1, C3), paired parasites were also observed (Figure 1, D3). Some of the parasites/organisms were positioned at the peripheries of the red blood cells (Figure 1, C3).

### 3.2. Molecular Detection of Babesia/Theileria in Cattle, Dogs and Humans

**Figure 2:**
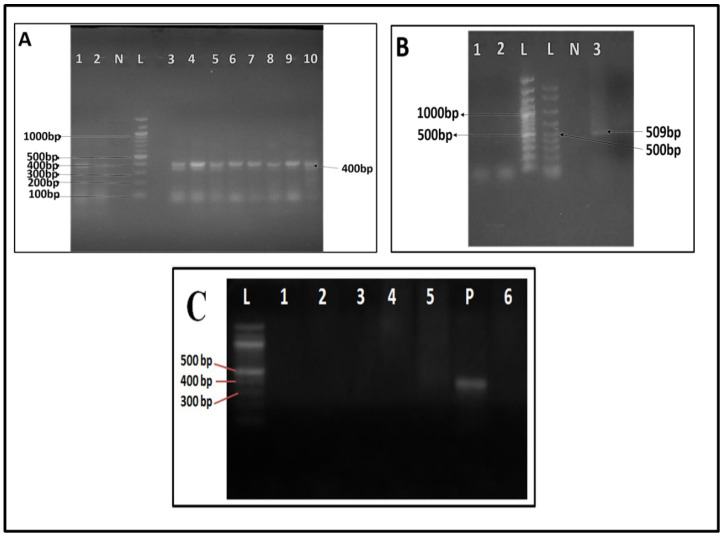
Agarose gels showing PCR results of samples from cattle (**A**), dogs (**B**) and humans (**C**) L: 100 base pair ladder. (**A**): Lanes 1, 3 - 10: PCR products showing band sizes of about 400 base pairs from amplification with *Babesia/Theilaria* primers, N—negative control, (**B**): Lanes 1–3 suspected dog samples, Lane 3—PCR amplification with *B. canis* primers; arrow shows a band size around 509 bp C: Lanes 1–6: suspected human samples after PCR showing no bands with the *Babesia/Theilaria* primers, P: positive control.

Gel electrophoresis of the PCR products revealed about 400 base pair bands in nine (9) out of the ten (10) suspected cattle samples (Figure 2A and Table 3). PCR amplification with *B. canis* primers on the sample from dog which showed features of haemoparasites, had a band size around 509 bp (Figure 2B). None of the suspected *Babesia/Theileria* samples from the humans as shown in Figure 1, Lane 3 was PCR-amplified (using *Babesia/Theileria* primers as well as the three other probable humans-associated *Babesia* primers), in this study (Figure 2C).

## 4. Discussion

The successful amplification of DNA from the cattle samples using primers that can detect some *Babesia* and *Theileria* species suggests that cattle in Accra might have some form of *Babesia*/*Theileria* infection as previously reported by Bell-Sakyi et al. [9]. Since the nine cattle samples in which amplification was successful with the *Babesia/Theileria* primer, did not have amplification with *B. bovis* and *B. divergens* (commonly known to infect cattle) primers, there is an urgent need for DNA sequencing to determine the genus (*Babesia* or *Theileria*) and species infecting these cattle. The importance of DNA sequencing is further highlighted in its ability to determine even to the strain level, as demonstrated by researchers in Korea, Washington and California, where detection of strains KO1, WA1 and CA1 were respectively observed [3,13,14].

The single cattle sample that was PCR-negative with the “*Babesia/Theileria* primers”, but showed intra-erythrocytic parasites, might have been infected with other species of *Babesia or Theileria* that were not tested for in this study, or even other pathogens. *Babesia bigemina*, which was not tested for in the current study, has been reported as infecting cattle in Ghana, with a prevalence of 6% [9]. *Theileria mutans*, *Theileria velifera* and *Anaplasma sp* occur as cytoplasmic inclusions in red blood cells. These organisms have also been reported to infect cattle in Ghana, with prevalence of 92%, 44% and 12% respectively [9]. Even though the cattle in this study were not likely to have been infected with *B. bovis* or *B. divergens*, the latter (*B. divergens*) which naturally infects cattle has been reported to cause most of the severe cases of human babesiosis in Europe [26]. Moreover, *B.* *bovis* was the species that caused illness in the first reported case of human babesiosis in 1956 [2]. Meanwhile, the *Babesia/Theileria* infecting these nine cattle could be of any type, species or strain (such as *B. occultans* and *B. major* which have been reported in Nigeria, a country close to Ghana) since the type of *Babesia* infection reported in Japan was described as different from the type in the United States, as well as those of the Kobe strain [27]. Again, a case reported in Korea showed that the strain detected in the patient was closely related to *Babesia sp* in sheep (ovine) than the species known to infect humans [3]. This suggests that the number of *Babesia* species infecting different hosts could be underestimated and that any species could be considered potentially zoonotic [27,28].

The successful PCR amplification with the *B. canis* primers from the sample of the dog could make this study among the few in the country that examined the infection in dogs using PCR [29]. Dogs have been known to be infected with *B. canis* complex [30], which has rarely been reported to cause disease in humans so far. There has, however, been a case of babesiosis in an Egyptian boy suspected to have contracted the infection from his pet dog [31], suggesting a link between dog-associated *Babesia* and human babesiosis. Moreover, about 37.6% of a study population in Mexico was reported to have antibodies against *B. canis* [28], suggesting that they had, at a point of their lives, been exposed to the parasite although, disease as a result of *B. canis* infection had not been reported. The disease had not manifested in these individuals probably because they were immune-competent, however, the same may not apply in immune-compromised individuals. Therefore, dog owners and keepers should ensure that ticks are well controlled on their dogs in order to minimise the risk of being bitten by ticks upon close contact with these pets.

The fact that among the human subjects in this study, none of the six “suspected” cases had amplification of the targeted genes of *Babesia/Theileria* with the primers used could be a good indication that human-vector contact might almost be negligible among the study population in the city. This observation could imply that individuals in Accra (being a city) seem to be careful not to be bitten by ticks even when they relate closely with pets and livestock that may have ticks on them; an observation which must be encouraged in the local community. In the meantime, the present study cannot draw conclusions relating to the prevalence of human piroplasms in the study sites.

## 5. Conclusions

This study has demonstrated detection of *Babesia*/*Theleria* in cattle and *B. canis* in a dog in Accra, but not in humans. The non-detection of *Babesia*/*Theleria* investigated in humans may be a good indication of negligible human-vector interaction in the city, Accra. However, the possibility of *Babesia*/*Theileria* infection in humans in other parts of the country cannot be overruled. It is therefore recommended that similar studies are done in other parts of Ghana to see the trend of *Babesia/Theileria* infection nationwide.

## 6. Limitations

DNA sequencing and phylogeny would have been useful in determining the type and strain of parasites involved in the infections recorded in this preliminary studies. This is considered a limitation, calling for the need for sequencing in future studies. Another limitation worth mentioning is the low quality of the images shown. The low quality is mainly because we had to improvise when taking the pictures, due to our inability to access high-quality image-acquiring systems. We admit that the picture quality is poor, and hence the need to emphasise the setting in which the study was conducted—a resource-limited one. However, for transparency about data obtained, we saw the need to present the images as we obtained them. We therefore recommend that, subsequently, a more advanced imaging system is used to obtain pictures of higher quality.

## Figures and Tables

**Table 1:** Primers used for amplification of PCR.

Primer Code (Name)	Oligonucleotide Sequence	Used for Detection of (Piroplasms)	Target Gene or Region	Size	Ref.
Bab5/ Bab8	AATTACCCAATCCTGACACAGG TTTCGCAGTAGTTCGTCTTTAACA	*Most Babesia*/*Theleria* spp. (including *B. microti*, *B. divergens* and *T. annae*)	18S rRNA	about 400 bp	[3,22]
Bab6/ Bab7	GACACAGGGAGGTAGTGACAAGA CCCAACTGCTCCTATTAACCATTAC				
BoF/ BoR	CACGAGGAAGGAACTACCGATGTTGA CCAAGGAGCTTCAACGTACGAGGTCA	*B. bovis*	rap-1	354 bp	[25]
ND	GTTTCTGMCCCATCAGCTTGAC CAATATTAACACCACGCAAAAATTC	Bovine *Babesia* spp. (including *B. divergens*)	18S rRNA	422–440 bp	[23]
BcW-A/ BcW-B	CATCTAAGGAAGGCAGCAGG TTAATGGAAACGTCCTTGGC	*B. canis*	18S rRNA	500 bp	[24]

ND: non-designated.

**Table 2:** Thermo-cycling conditions used in the amplification of the other species.

Primers	Denaturation	Annealing	Extension	Cycles	Fragment Size
*B. divergens*	94 °C (30 s)	61 °C (30 s)	72 °C (45 s)	35	353 bp
*B canis*	94 °C (2 min)	60 °C (30 s)	72 °C (30 s)	35	509 bp
*B. bovis*	94 °C (30 s)	63 °C (30 s)	72 °C (1 min)	35	350 bp

min represents minutes, s represents seconds, bp represents base pair.

**Table 3:** Outcome of microscopy and polymerase chain reaction (PCR) examinations for the various hosts.

Host	N	“Suspected” Based on Microscopy *n* (%)	Amplification of “Suspected” by PCR, *n* (%)			
			*Babesia/Theileria*^#^ N * n *	*B. bovis* ^#^ N * n *	*B. divergens* ^#^ N * n *	*B. canis* ^#^ N * n *
Cattle	30	10 (33)	9 (30) 9 (90)	0 (0) 0 (0)	0(0) 0 (0)	NS NS
Dogs	33	3 (9)	0 (0) 0 (0)	NS NS	NS NS	1 (3) 1 (33)
Humans	150	6 (4)	0 (0) 0 (0)	0 (0) 0 (0)	0 (0) 0(0)	0 (0) 0 (0)

N = total number of samples taken for each host; *n* represents the number positive; N * = the denominator used was the total number sampled for each subject, n * = the denominator used was the number of suspected for each of the subjects, NS = sample not screened with this primer; **^#^** = primers for the indicated organism.

## Data Availability

All data supporting reported results have been included in paper.

## Sample Availability

Samples of the compounds are available from the authors.

## Abbreviations

A: adenine, B.: *Babesia,* bp: base pair, C: cytosine, DNA: deoxyribonucleic acid, G: guanine, min: minute(s), *P*. *falciparum*: *Plasmodium falciparum*, Pf HRP-2: *Plasmodium falciparum* histidine rich protein 2, UGMS: University of Ghana Medical School, T: thymine, Taq: *Thermus aquaticus,* μL: micro litre, μm: micro metre, μM: micro molar. EDTA: Ethylenediaminetetraacetic acid, pLDH: Plasmodium lactate dehydrogenase, PCR: polymerase chain reaction.
